# Basic patches on the E2 glycoprotein of eastern equine encephalitis virus influence viral vascular clearance and dissemination in mice

**DOI:** 10.1128/jvi.00602-25

**Published:** 2025-05-19

**Authors:** Stephanie E. Ander, Erin R. Fish, Mariana O. L. da Silva, Bennett J. Davenport, M. Guston Parks, Thomas E. Morrison

**Affiliations:** 1 Department of Immunology and Microbiology, University of Colorado School of Medicine 12225, Aurora, Colorado, USA; 2 Department of Microbiology and Immunology, Louisiana State University Health Shreveport 440927 , Shreveport, Louisiana, USA; 3 Instituto de Microbiologia Paulo de Goes, Universidade Federal do Rio de Janeiro 28125, Rio de Janeiro, Brazil; St. Jude Children's Research Hospital, Memphis, Tennessee, USA

**Keywords:** *Alphavirus*, viremia, EEEV, glycosaminoglycan, heparan sulfate

## Abstract

Previously, we found that chimeric Sindbis-eastern equine encephalitis virus (SINV-EEEV) particles can be removed from the murine blood circulation in a phagocyte-dependent manner which can be disrupted by either transient depletion of vascular heparan sulfate (HS) glycosaminoglycans (GAGs), or mutation of the viral E2 glycoprotein (K71/74/77A) associated with decreased GAG binding *in vitro*. Here, we further investigate the viral determinants of EEEV vascular clearance and evaluate their role in viremia development. We identified two large basic patches on the EEEV E2 glycoprotein which contain two known GAG-binding sites (K71/74/77 and K156/R157) and six additional basic residues (K10, R13, K56, R152, K231, and K232). We find that disruption of either basic patch by single alanine substitutions promotes prolonged retention of SINV-EEEV particles in the murine blood circulation in an experimental viremia model. Furthermore, we observed that the K156/R157A, K10A, and K231A mutations are also associated with similar viral dissemination in a mouse infection model as the attenuated K71/74/77A mutant. Surprisingly, despite known differences in GAG binding and potential alteration in receptor interactions, we find the initial dispersal of wild-type (WT) and mutant SINV-EEEV virions from the inoculation site to the draining lymph node to be equivalent at 1 hour post-subcutaneous inoculation. Moreover, our data suggest the higher viremia associated with mutation of the E2 basic patches may be attributed to evasion of viremic control by blood-filtering phagocytes. Overall, this study defines viral features of the EEEV E2 glycoprotein that influence tissue-specific viral dissemination and highlights the capacity of blood-filtering phagocytes to modulate EEEV viremia.

**IMPORTANCE:**

Virus-GAG interactions have long been studied *in vitro*; however, investigating the impact of these interactions *in vivo* has been challenging. Previously, we showed that blood-filtering phagocytes and vascular HS mediate the removal of enhanced GAG-binding WT SINV-EEEV virions from the blood circulation in a reductionist, experimental viremia model. Here, we demonstrate that single-residue, charge-neutralizing mutations within basic patches of the E2 glycoprotein are sufficient both to promote viral evasion of vascular clearance and viral dissemination in an infection model. We also find that the WT and decreased GAG-binding SINV-EEEV virions traffic similarly from a subcutaneous inoculation until drainage into the bloodstream, upon which the WT virus is selectively depleted. These observations suggest viral dissemination is influenced by tissue-specific, virion-GAG interactions.

## INTRODUCTION

Eastern equine encephalitis virus (EEEV) is an arthropod-borne virus (arbovirus) in the *Alphavirus* genus and persists in nature in transmission cycles between avians and mosquitoes. However, spillover of EEEV into human and equine populations can be associated with devastating disease. At least 33% of reported human cases are lethal, and survivors suffer permanent neurological sequelae; no approved vaccine or specific antiviral treatment to prevent or treat EEEV infection exists (1).

Natural isolates of EEEV are particularly noted for their virulence despite enhanced binding to glycosaminoglycans (GAGs) *in vitro*, and mutations in the virus that disrupt GAG interactions are attenuating *in vivo* (2, 3). GAGs are linear polysaccharides ubiquitously present at cell surfaces and the extracellular matrix that function in a variety of different biological processes, including establishment of chemokine gradients, cell signaling and growth, and angiogenesis (4). GAGs such as heparan sulfate (HS) are known to promote viral attachment through electrostatic interactions between their negatively charged, sulfated sugars and basic amino acids on the virion surface (2–5). Among arboviruses, enhanced GAG-binding phenotypes may easily arise through cell culture adaptations (5); these mutations are often associated *in vivo* with enhanced clearance of those virions from the blood circulation (6–10), less efficient viral dissemination (2, 6, 9, 11–15), and attenuation of disease (8, 11, 13, 15–18). However, some natural and low-passage arbovirus isolates also exhibit affinity for GAGs, such as EEEV, Venezuelan equine encephalitis virus, chikungunya virus (CHIKV), dengue virus, and Rift Valley fever virus (2, 3, 19–24).

Previous studies demonstrated a role for HS interactions in promoting wild-type (WT) EEEV infection *in vitro* (2, 3), and EEEV virions bound to HS have also been visualized by cryo-electron microscopy (cryo-EM) (25). These studies identified three regions on the viral E2 glycoprotein involved in mediating virion-HS interactions *in vitro*: amino acid residues K71/74/77 (2, 3), K156/R157 (25), and, to a lesser extent, R84/119 (25). Recently, some of these regions were shown to also interact with the cellular protein receptors VLDLR and ApoER2 (residues K156/R157) or to be located near the receptor-binding sites (K71/74/77) (26–28). A recent preprint also supports this overlap of HS- and protein receptor-binding sites, wherein viral residues required for HS interactions may promote receptor-mediated infection directly (K156/R157) or indirectly (K71/74/77) (29).

Using an experimental model of viremia, we previously demonstrated that WT particles of SINV-EEEV, a higly attenuated chimeric virus encoding the Sindbis virus (SINV) nonstructural proteins and RNA replication regulatory sequences along with the EEEV structural proteins, are efficiently removed from the blood circulation of mice, while the K71/74/77A mutation is sufficient to disrupt this vascular clearance phenotype (10). In addition, vascular clearance of circulating WT SINV-EEEV particles is sensitive to transient depletion of blood-filtering phagocytes and vascular HS (10). We also observed species-level specificity in SINV-EEEV vascular clearance, as viral vascular clearance selectively occurred in a murine but not an avian model of viremia (models of dead-end and amplifying hosts, respectively, for EEEV) (10).

Here, we further elucidate features of the WT SINV-EEEV virion required for viral vascular clearance and investigate the impact of these viral determinants on viral dissemination in a murine infection model. We find charge ablation by alanine substitution of the three individual known HS interaction sites (K71/74/77, K156/R157, and R84/119) to be sufficient to disrupt SINV-EEEV vascular clearance in an experimental viremia model. Applying a structure-guided approach focused on the K71/74/77 and K156/R157 sites, we identified that these sites are constituents of two larger basic patches on the EEEV E2 glycoprotein. Disruption of these basic patches by alanine substitution of other basic residues (K10, R13, K56, R152, K231, and K232) is similarly sufficient to disrupt SINV-EEEV vascular clearance. Furthermore, disruption of either basic patch is associated with enhanced viremia and greater viral burden in the brain after 1 day following subcutaneous (s.c.) inoculation with SINV-EEEV. Examining the initial dispersal of inoculated virions, we found that the WT and K71/74/77A mutant traffic similarly to the draining lymph node. However, only WT SINV-EEEV viremia is modulated in a phagocyte-dependent manner. Overall, this study defines SINV-EEEV virion features important for mediating putative GAG interactions *in vivo*, and it provides further insight into the role of tissue-specific, virion-GAG interactions in viral dissemination.

## RESULTS

### Defining the role of known viral determinants of EEEV-HS binding for *in vivo* viral vascular clearance

**Fig 1:**
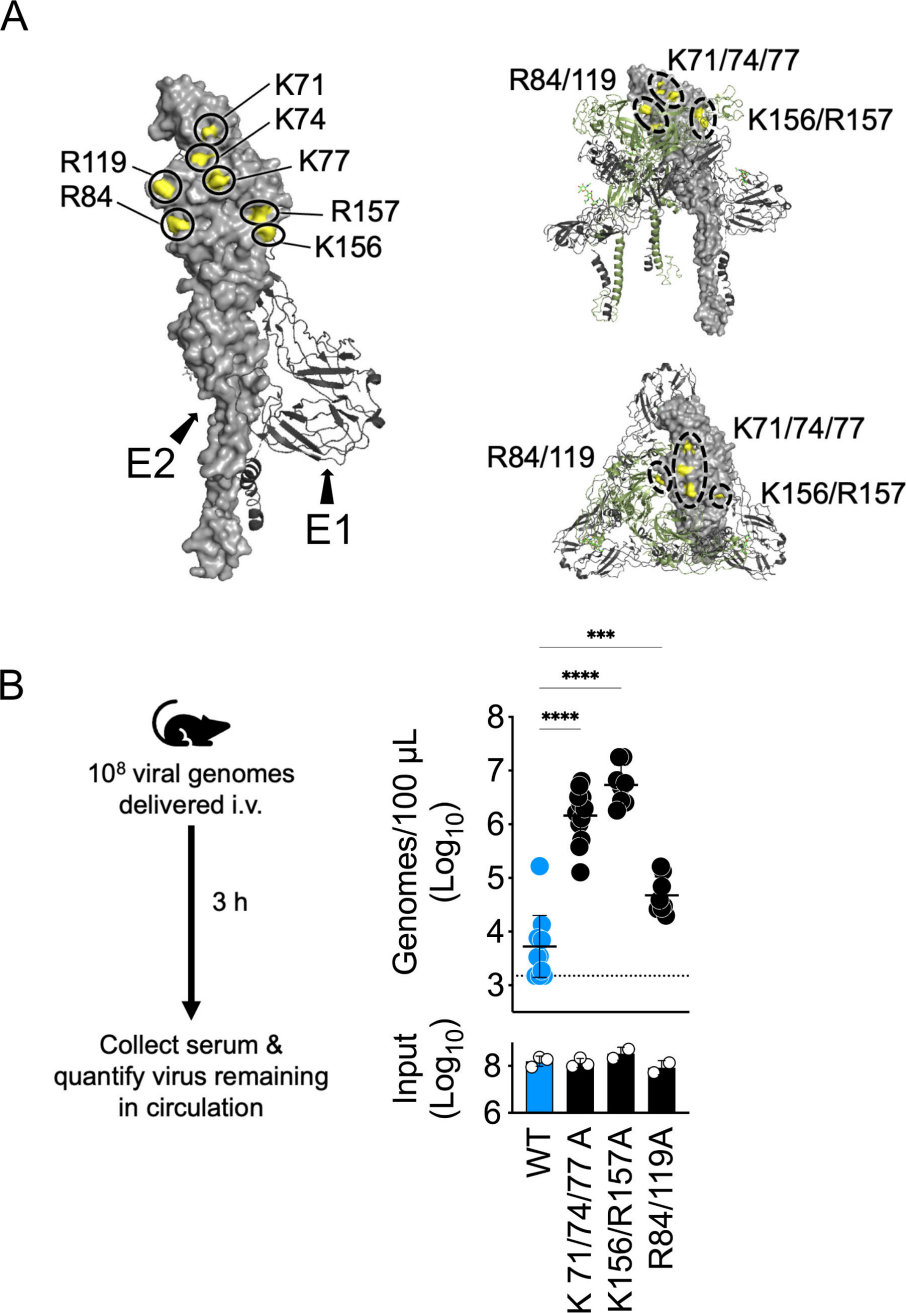
SINV-EEEV E2 glycoprotein mutations known to disrupt HS binding *in vitro* are associated with impaired viral vascular clearance in an experimental viremia model. (**A**) PyMOL rendering of EEEV E2-E1 glycoprotein heterodimer (PDB structure 6m×4 [31]) with labels indicating positions of E2 residues previously known to interact with HS *in vitro* (left). Also shown are the positions of these HS-interacting residues in the context of the virion spike (right); a trimer of E2-E1 heterodimers (additional heterodimers that make up the spike are illustrated as ribbon diagrams where E2 is colored green). (Top right) Side view of EEEV spike; (bottom right) top-down view of EEEV spike. (**B**) Design of the experimental viremia model used to assess virion-host interactions in the vasculature (left). Vascular clearance of SINV-EEEV particles from murine circulation at 3 hpi (right). WT C57BL/6 mice were inoculated i.v. with 10^8^ genomes (input); blood was collected at 3 hpi, and viral genomes in the serum were quantified. Data are from two to three independent experiments, four to five mice per virus in each experiment, and graphed to display the mean and the standard deviation for each group. Statistics determined by one-way analysis of variance followed by Dunnett’s multiple comparison test. ****, *P* < 0.0001; ***, *P* < 0.001.

Previous studies on EEEV identified three regions on the viral E2 glycoprotein that promote virion binding to HS *in vitro* (Fig. 1A): lysines-71/74/77 (2, 3), lysine-156/arginine-157 (25), and arginines-84/119 (25). We investigated the importance of these same residues for clearance of SINV-EEEV virions from the blood circulation, which we previously showed is mediated by blood-filtering phagocytes and vascular HS (10), using an *in vivo* experimental viremia model (Fig. 1B). To permit investigations of EEEV virion-host interactions at BSL-2 containment, we utilized a chimeric Sindbis virus expressing the structural proteins of EEEV which produces attenuated virus structurally similar to authentic EEEV virions (30). We applied these SINV-EEEV particles (measured in viral genomes) to our mouse model of experimental viremia, wherein C57BL/6 mice are intravenously (i.v.) inoculated with a high titer of virus, and then we quantified the number of virus genomes remaining in the circulation at 3 hours post-inoculation (hpi) (Fig. 1B).

**Fig 2:**
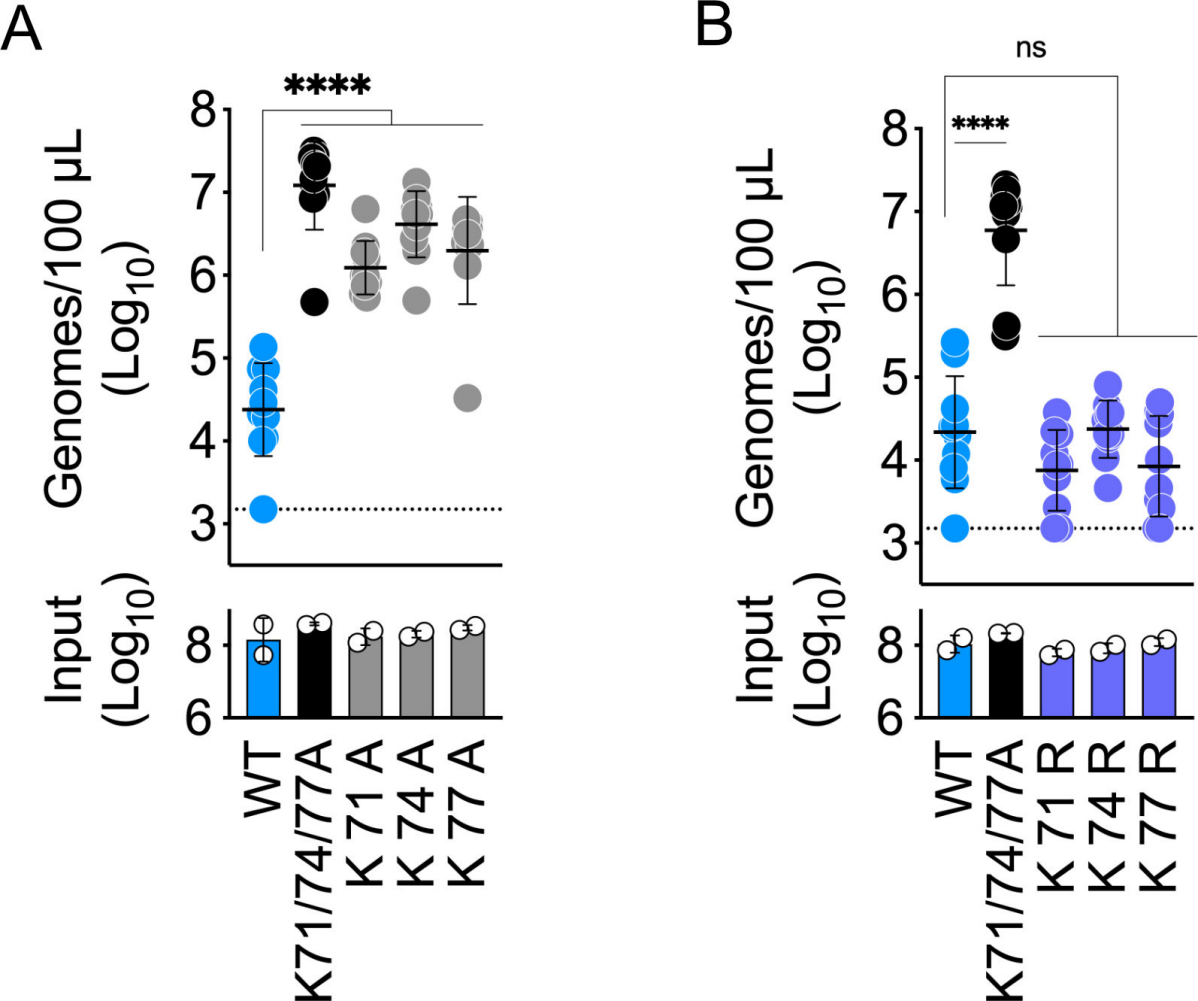
Presence of a basic charge at E2 glycoprotein positions 71, 74, and 77 is required for SINV-EEEV vascular clearance. Vascular clearance of SINV-EEEV particles from murine circulation at 3 hpi. WT C57BL/6 mice were inoculated i.v. with 10^8^ genomes (input); blood was collected at 3 hpi, and viral genomes in the serum were quantified. (**A**) Comparison of individual alanine substitutions at E2 glycoprotein positions 71, 74, or 77 against WT and K71/74/77A triple mutant. (**B**) Comparison of individual arginine substitutions at E2 glycoprotein positions 71, 74, or 77 against WT and K71/74/77A triple mutant. Data are from two independent experiments, five mice per virus in each experiment, and graphed to display the mean and the standard deviation for each group. Statistics determined by one-way analysis of variance followed by Dunnett’s multiple comparison test. ****, *P* < 0.0001. ns, not significant.

In accordance with our previously published observations on SINV-EEEV interactions with vascular HS (10), we found that WT SINV-EEEV particles are efficiently removed from the circulation by 3 hpi (Fig. 1B). In contrast, both the K71/74/77A and the K156/R157A ∆HS-binding mutants persist at a high concentration in the circulating blood at 3 hpi (123- and 406-fold higher than WT, respectively) (Fig. 1B). The R84/119A mutations resulted in less efficient vascular clearance with 3.4-fold more virus persisting in the serum at 3 hpi than WT (Fig. 1B). Further investigation of the three Lys residues at positions 71, 74, and 77 found that single alanine substitution at any one of these three positions was sufficient to disrupt SINV-EEEV vascular clearance (Fig. 2A). In contrast, amino acid substitution at any one of these three positions with another basic residue, arginine, did not alter viral vascular clearance in comparison with WT SINV-EEEV (Fig. 2B), suggesting that a positive charge at each of these sites is critical for clearance of SINV-EEEV virions from the blood circulation.

### Preservation of two basic patches on the E2 glycoprotein is required for SINV-EEEV vascular clearance

**Fig 3:**
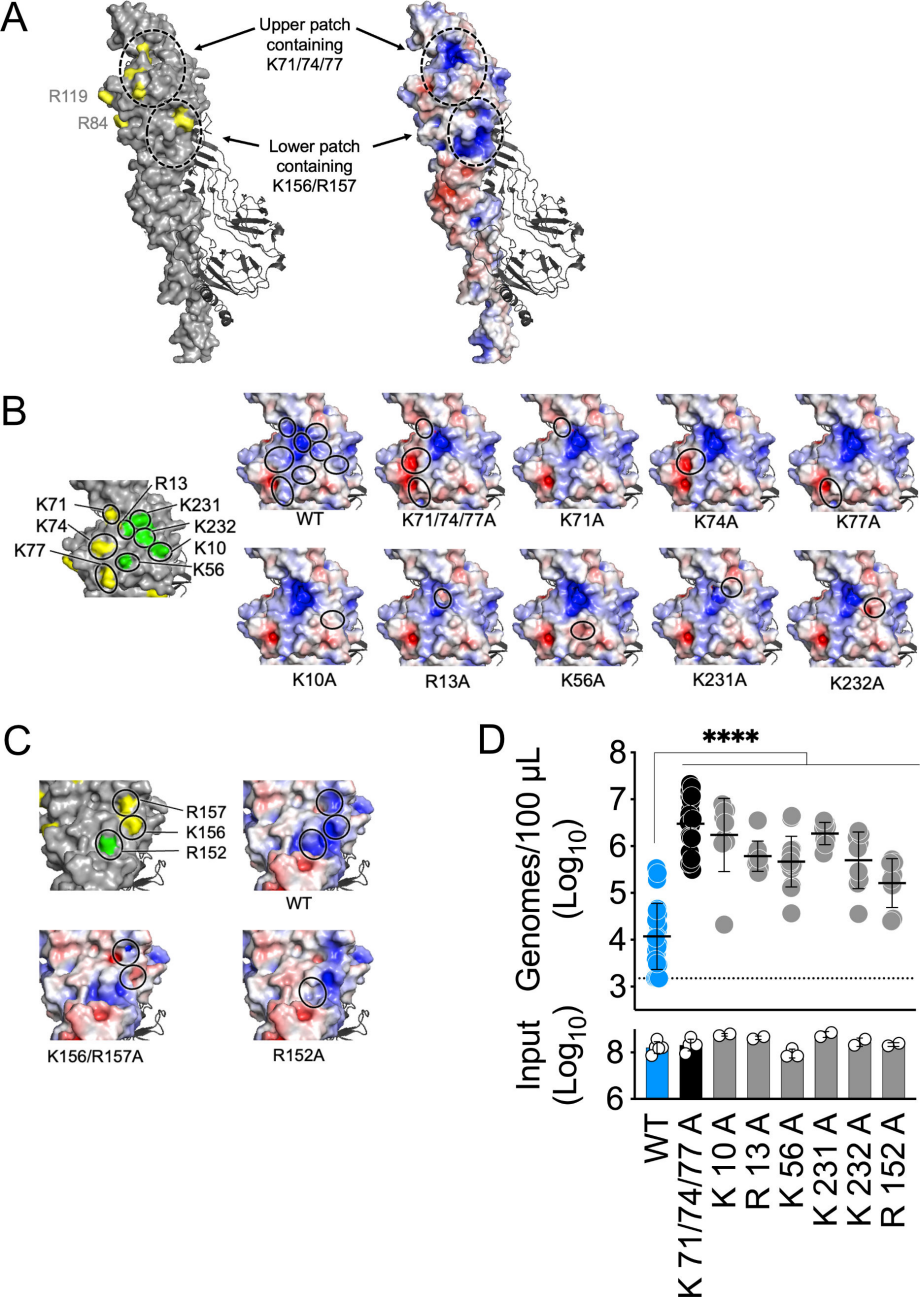
Preservation of basic patches on the E2 glycoprotein is required for SINV-EEEV vascular clearance. (**A**) 3D rendering of WT EEEV E2-E1 glycoprotein heterodimer (PDB structure 6mx4 [31]) with residues previously shown to mediate interactions with HS highlighted in yellow (left) as well as an electrostatic map of the surface charge distribution. (**B**) Zoom-in of upper basic patch. (**C**) Zoom-in of lower basic patch. (**B and C**) Additional residues selected for investigation are highlighted in green. Also shown are the predicted changes to electrostatic potential on E2 glycoprotein with each indicated alanine substitution; location of the mutated residue is indicated with a circle. (**D**) Vascular clearance of WT and mutant SINV-EEEV particles from murine circulation at 3 hpi. WT C57BL/6 mice were inoculated i.v. with 10^8^ genomes (input); blood was collected at 3 hpi, and viral genomes in the serum were quantified. Data are from at least two independent experiments, four to five mice per virus in each experiment, and graphed to display the mean and the standard deviation for each group. Statistics determined by one-way analysis of variance followed by Dunnett’s multiple comparison test. ****, *P* < 0.0001.

Given that HS-protein interactions are mediated by electrostatic interactions between negatively charged HS and positively charged regions on the interacting protein, we generated an electrostatic map of the WT E2-E1 EEEV heterodimer (Fig. 3A). This analysis revealed that the K71/74/77 and the K156/R147 HS interaction sites are constituents of two larger, encompassing basic patches on the E2 glycoprotein (which we refer to as the upper and lower basic patches, respectively). Thus, we hypothesized that individual alanine substitutions targeting other positively charged residues within these basic patches (K10, R13, K56, K231, K232, and R152) would influence the vascular clearance of WT SINV-EEEV virions. PyMOL predictive modeling of each alanine substitution suggests these mutations alter the electrostatic potential of either the upper or lower basic patches (Fig. 3B and C). We next generated this panel of mutant viruses and assessed their vascular clearance phenotypes in our *in vivo* model of experimental viremia. As hypothesized, we observed that these other individual alanine substitutions targeting the upper (K10A, R13A, K56A, K231A, and K232A) and lower (R152A) E2 basic patches are also sufficient to significantly disrupt SINV-EEEV vascular clearance (Fig. 3D). In contrast, the charge-preserving mutation K56R does not affect SINV-EEEV vascular clearance (Fig. S1). These data show that all positively charged residues comprising the upper (K10, R13, K56, K71, K74, K77, K231, and K232) and lower (K156/R157 and R152) basic patches of the EEEV E2 glycoprotein are critical determinants of the efficient clearance of SINV-EEEV virions from the blood circulation.

### SINV-EEEV E2 glycoprotein basic patch mutants resistant to viral vascular clearance are associated with enhanced viremia following subcutaneous inoculation

**Fig 4:**
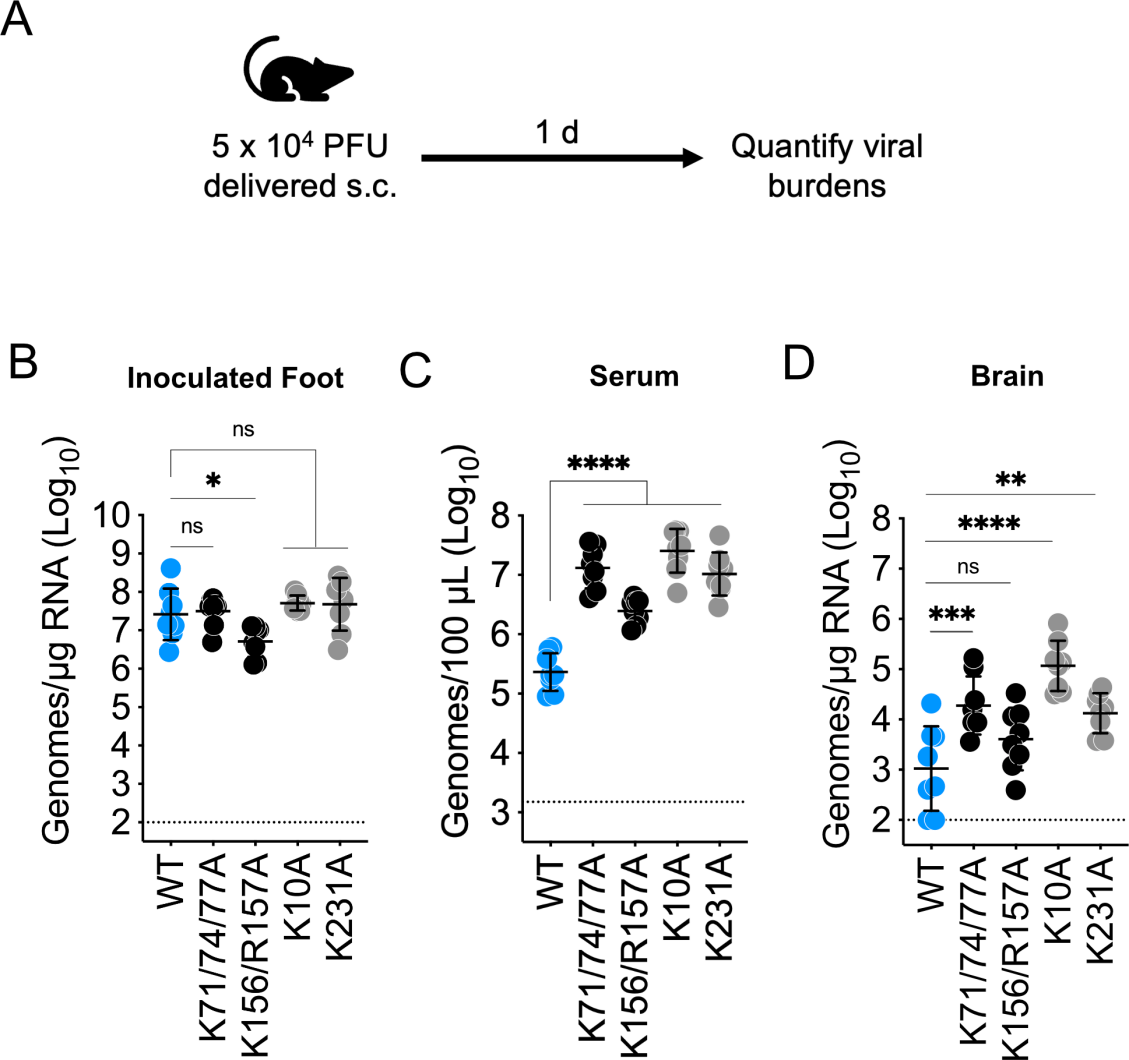
SINV-EEEV E2 glycoprotein mutations associated with disrupted interactions with vascular HS promote dissemination following s.c. inoculation of mice. WT C57BL/6 mice were inoculated with 5 × 10^4^ PFU of SINV-EEEV in a 20 µL volume by s.c. inoculation into the left rear footpad (**A**). At 1 dpi, virus burdens were quantified by reverse transcription quantitative PCR at the site of inoculation (**B**), in the serum (**C**), and in the brain (**D**). Data are from two independent experiments, four mice per virus in each experiment, and graphed to display the mean and the standard deviation for each group. Statistics determined by one-way analysis of variance followed by Dunnett’s multiple comparison test. ****, *P* < 0.0001; ***, *P* < 0.001; **, *P* < 0.01; *, *P* < 0.05. ns, not significant.

We next investigated the impact of E2 glycoprotein basic patch mutations on viral dissemination following s.c. inoculation into WT C57BL/6 mice (Fig. 4A). In addition to WT SINV-EEEV, we included four of the E2 glycoprotein mutants that displayed the greatest resistance to viral vascular clearance (mutants K71/74/77A, K156/R157A, K10A, and K231A; Fig. 3D). At 1 day post inoculation (dpi), we observed minimal differences in virus burden at the site of inoculation, within 0.09- to 1.2-fold the burden of WT (Fig. 4B), indicating these mutations do not alter viral replication *in vivo*. However, all four mutants exhibited significantly enhanced viremia at 1 dpi compared with WT: 58.8-fold viral RNA burden in the serum for K71/74/77A, 9.4-fold for K156/R157A, 112.3-fold for K10A, and 49.5-fold for K231A (Fig. 4C). A similar trend was observed when comparing infectious virus titers of these serum samples, with E2 glycoprotein mutations K10A and K231A associated with significantly enhanced infectious viremia (Fig. S2). As authentic EEEV is neuroinvasive, we also assessed whether these E2 basic patch mutations affected SINV-EEEV burdens in the brain. We found elevated viral RNA burdens in the brain for most of the mutant viruses: K71/74/77A was present at 10.5-fold higher levels than WT; K10A was enhanced 51.5-fold; and K231A was 4.4-fold enhanced (Fig. 4D). Notably, the K156/R157A mutant virus did not exhibit any significant difference in viral RNA burden in the brain at 1 dpi when compared against WT SINV-EEEV (2.1-fold, *P*_adj_ = 0.1821).

### WT and K71/74/77A SINV-EEEV particles disperse similarly from the s.c. inoculation site to the draining lymph node, but WT virions are selectively removed by blood-filtering phagocytes upon drainage into the circulating blood

**Fig 5:**
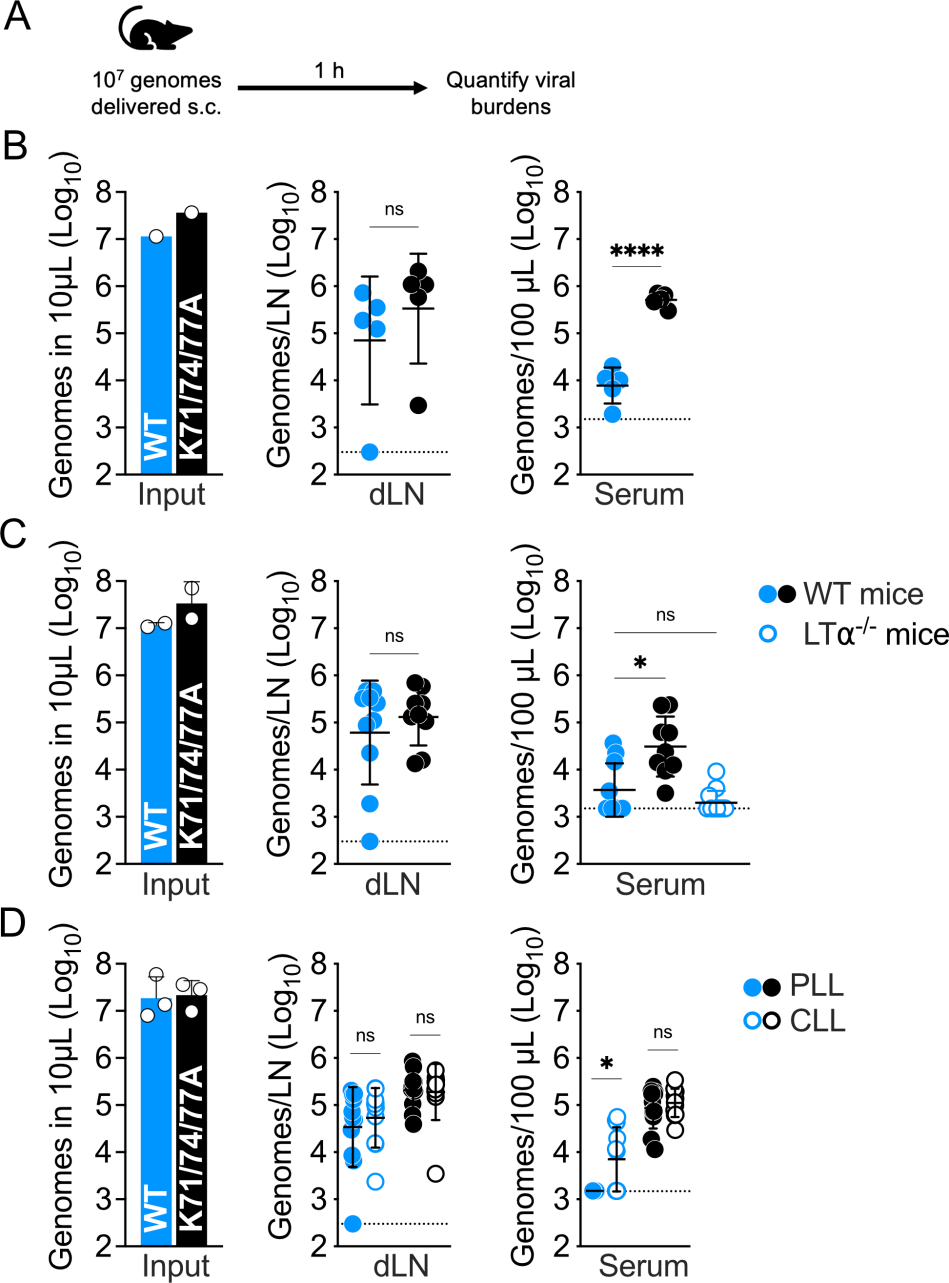
WT and K71/74/77A SINV-EEEV disperse similarly from the s.c. inoculation site to the draining lymph node but differ in serum viral loads at 1 hpi. (**A–D**) To study the initial dispersal of inoculated virions, mice were inoculated s.c. in the left rear footpad with 10^7^ SINV-EEEV genomes in a 10 µL volume (input), and viral loads in the draining lymph node (dLN) and serum at 1 hpi were determined by reverse transcription quantitative PCR. (**B**) Dispersal of WT and K71/74/77A SINV-EEEV inoculum in WT C57BL/6 mice. (**C**) Comparison of SINV-EEEV dispersal in WT mice and mice lacking peripheral lymph nodes (LTα^−/−^ mice). (**D**) At −2 dpi, WT mice were treated with clodronate-loaded liposomes (CLL) or phosphate-buffered saline-loaded liposomes (PLL) (control) to deplete blood-filtering phagocytes and assess the impact on SINV-EEEV dispersal at 1 hpi. Data are from one three independent experiments, three to five mice per virus in each experiment, and graphed to display the mean and the standard deviation for each group. Statistics determined by unpaired Student’s *t*-test (**B**, serum and C-dLN), Mann-Whitney test (**B**, dLN; **D**), or Kruskal-Wallis test followed by Dunn’s multiple comparison test (**C**, serum). ****, *P* < 0.0001; *, *P* < 0.05. ns, not significant.

We hypothesized that differences in viremia development following s.c. inoculation may be related to the initial dispersal of virions from the site of inoculation. Specifically, we expected the enhanced GAG-binding WT virions would be more likely to be trapped locally at the site of inoculation and would be present to a lesser degree at the draining lymph node and in the circulating blood than the K71/74/77A mutant, which exhibits decreased interactions with HS (2). To test this idea, we s.c. inoculated 10^7^ particles (measured by viral genomes) into the left rear footpad, then quantified viral burden in the draining (popliteal) lymph node and the serum at 1 hpi (Fig. 5A). Surprisingly, we found WT and K71/74/77A were present in the draining lymph node at similar levels; however, there was significantly more K71/74/77A virus (53-fold) in the circulating blood at this same early timepoint (Fig. 5B). These data suggest that the enhanced GAG-binding properties of WT SINV-EEEV virions do not affect the initial dispersal of inoculated virions from the s.c. inoculation site to the draining lymph node. Additional experiments found similar low levels of WT virus in the serum of both WT and lymphotoxin-alpha-deficient mice (which fail to develop peripheral lymph nodes [32]) (Fig. 5C).

We hypothesized the WT virus (but not K71/74/77A) may be selectively depleted upon drainage into the blood circulation by blood-filtering phagocytes, similar to our previous observations in the experimental viremia model (10). We tested this idea by pretreating mice i.v. with phosphate-buffered saline-loaded liposome (PLL) or clodronate-loaded liposomes (CLLs) at −2 dpi to deplete blood-filtering phagocytes, and we then quantified viral accumulation in the serum at 1 hpi following s.c. inoculation. We found that depletion of blood-filtering phagocytes was associated with a greater likelihood of detectable WT virus in the serum, while there was no effect on the K71/74/77A mutant serum levels (Fig. 5D). Phagocyte depletion also did not affect the levels of either virus in the draining lymph node at 1 hpi (Fig. 5D). These data suggest that the enhanced GAG-binding properties of WT SINV-EEEV virions do not affect the initial dispersal of inoculated virions from the site of inoculation to the draining lymph node and drainage therefrom to the blood. However, upon reaching the blood, s.c. inoculated SINV-EEEV is selectively depleted by blood-filtering phagocytes in a manner reliant on preservation of known GAG-binding sites on the virion surface.

### Blood-filtering phagocytes regulate SINV-EEEV viremia

**Fig 6:**
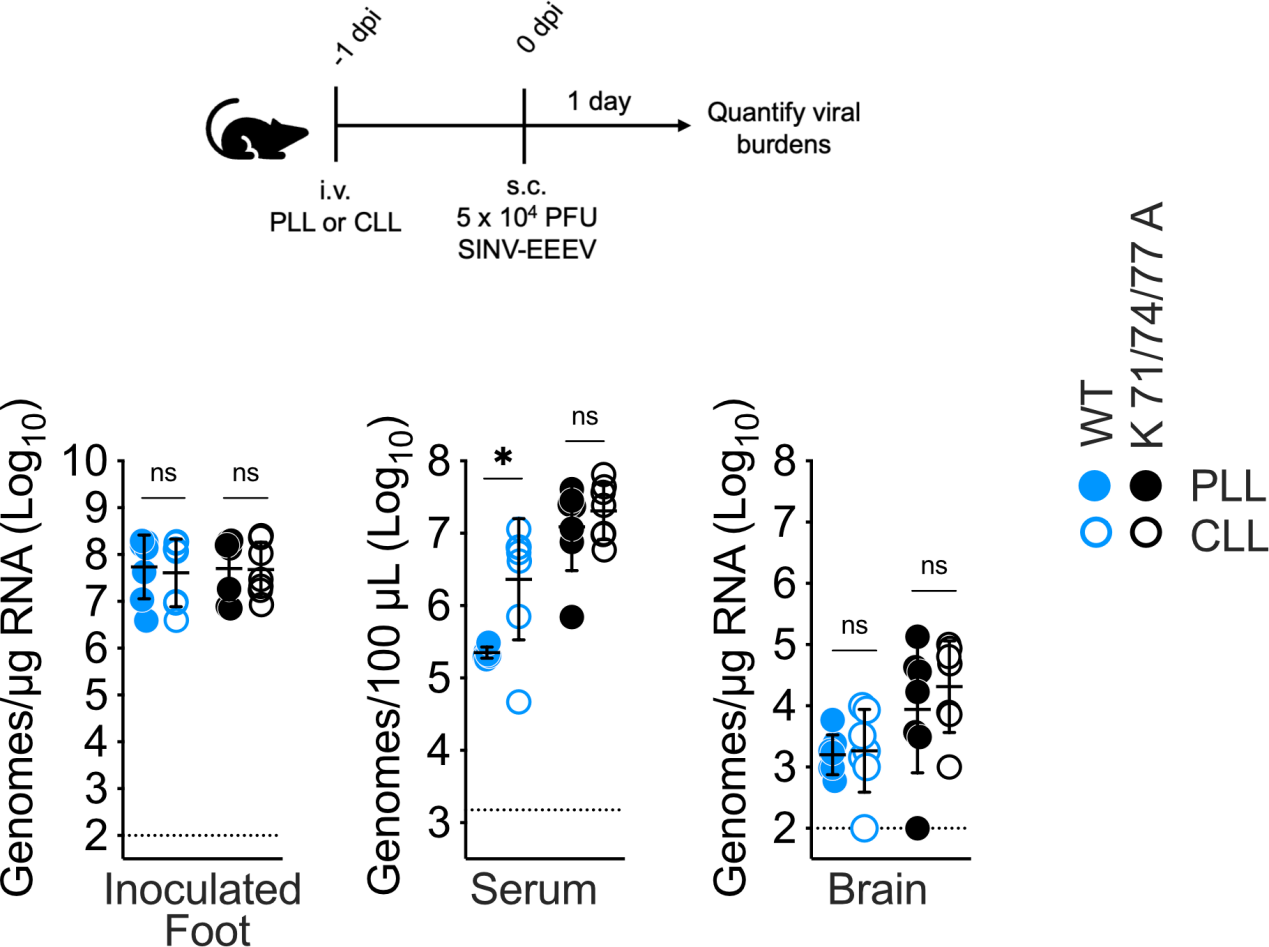
Blood-filtering phagocytes regulate SINV-EEEV viremia. At −1 dpi, WT mice were treated with CLL or PLL (control) to deplete blood-filtering phagocytes. Mice were then s.c. inoculated with 5 × 10^4^ PFU of SINV-EEEV WT or K71/74/77A mutant in a 20 µL volume into the left rear footpad, and viral burdens at 1 dpi were quantified in different tissues. Data are from two independent experiments, three to four mice per virus in each experiment, and graphed to display the mean and the standard deviation for each group. Statistics determined by unpaired Student’s *t*-test (brain) or Mann-Whitney test (inoculated foot, serum). *, *P* < 0.05. ns, not significant.

Having identified a role for blood-filtering phagocytes in specifically depleting WT SINV-EEEV from the bloodstream early after s.c. inoculation (Fig. 5D), we next assessed whether these cells may also affect viral dissemination at 1 dpi in an infection model. As empty phagocyte niches can quickly become repopulated (33), we modified our phagocyte depletion protocol to ensure continued effective depletion by 1 dpi. For these experiments, mice were pretreated i.v. with PLL or CLL and inoculated with the virus 24 hours later. To determine the effectiveness of CLL treatment after 24 hours, CHIKV, Ross River virus, and SINV-EEEV vascular clearance was assessed. Similar to our previous reports (10, 34), we observed a significant inhibition of vascular clearance for all three viruses in CLL- versus PLL-treated mice at 1 hpi (1 day after liposome treatment, Fig. S3). Having thus validated the effectiveness of CLL by 1 day after treatment, we then assessed the impact of blood-filtering phagocyte depletion on SINV-EEEV dissemination at 1 dpi. Phagocyte depletion did not affect WT and K71/74/77A viral burdens at the site of inoculation (Fig. 6). However, WT levels in the serum were significantly enhanced in phagocyte-depleted mice (21.9-fold), while the CLL pretreatment had no effect on K71/74/77A viremia (Fig. 6). Notably, while phagocyte depletion enhanced WT viremia, there was no similar enhancement of viral load in the brain, and the K71/74/77A mutant remained present at a higher burden in the brain than WT even in CLL-treated mice (Fig. 6).

## DISCUSSION

Due to the small volume of blood taken by the mosquito during a blood meal, viremia development in vertebrate hosts is critical for the transmission of EEEV and other mosquito-borne viruses. Viremia development is a complex process that can be regulated at multiple stages during infection, including through interactions of the virion with the host vasculature (35). To investigate determinants of alphavirus viremia restriction in the blood circulation, we have utilized a reductionist, experimental viremia model wherein animals are inoculated intravascularly (i.v.) with a high dose of virus to simulate the viremic state, and viral vascular clearance is assessed. We previously used this model to identify roles for the scavenger receptor MARCO and liver Kupffer cells in controlling viremia of arthritogenic alphaviruses CHIKV, Ross River virus, and o’nyong’nyong virus, as well as disease severity (34, 36, 37). We also applied this model to investigate the role of vascular GAGs in viremia modulation using low and enhanced GAG-binding viral variants of SINV-EEEV, SINV-VEEV, and CHIKV (10). We found that transient depletion of vascular HS *in vivo* interferes with phagocyte-mediated vascular clearance of enhanced GAG-binding SINV-EEEV and CHIKV virions in a mouse experimental viremia model (10).

Here, our initial experiments focused on the three known GAG-binding sites on the EEEV virion (all located on the E2 glycoprotein): K71/74/77, K156/R157, and R84/119 (2, 3, 25). Despite their attenuating effects on neurovirulence (2, 3, 29), alanine substitution of the K71/74/77 and the K156/R157 sites produced viral particles that persisted at high levels in the blood circulation for at least 3 hpi in the experimental viremia model (Fig. 1B). Meanwhile, the R84/119A mutation produced a less efficient, intermediate vascular clearance phenotype (Fig. 1B). As the R84/119 site was previously associated with weaker GAG interactions *in vitro* (25) and is located within a central pocket at the interface of the three E2-E1 heterodimers that oligomerize to form the viral spike protein (Fig. 1A), we hypothesize this intermediate vascular clearance phenotype may be due to limited accessibility of this GAG-binding site. In contrast, the K71/74/77- and K156/R157 GAG-binding sites are constituents of two larger, surface-exposed basic patches on the EEEV E2 glycoprotein (Fig. 3A). Single alanine substitutions of positively charged residues within these basic patches (K10, R13, K56, K71, K74, K77, K231, K232, and R152) were sufficient to produce similar vascular persistence phenotypes as the K71/74/77A mutant (Fig. 2A and 3D). These individual alanine substitutions are not expected to significantly disrupt viral protein folding, as it has been previously shown that monoclonal antibodies recognizing the B domain of the EEEV E2 glycoprotein bind similarly to WT and individual alanine point mutations of the residues investigated herein (38). Notably, a previous report on the K71/74/77 HS-binding site suggests alanine substitution of K77A does not disrupt HS interactions *in vitro* (3). However, our *in vivo* data indicate that positively charged amino acids at all three positions are required for viral vascular clearance (Fig. 2). As a similar dependence on amino acid charge was observed at position 56 (Fig. S1), we propose that SINV-EEEV interactions with vascular GAGs *in vivo* (10) are mediated by preservation of these basic patches rather than specific viral residues.

We also investigated the impact of a subset of E2 basic patch mutants in a mouse model of viral dissemination. In agreement with a previous study using authentic EEEV and despite attenuating neurovirulence (2), we observed the K71/74/77A mutation enhanced the viremia and viral brain burdens of the SINV-EEEV chimera in a natural infection model (s.c. inoculation, Fig. 4). Also, as seen with our experimental viremia model, we found blood-filtering phagocytes to be important regulators of WT, but not K71/74/77A, SINV-EEEV viremia at 1 day following s.c. inoculation (Fig. 6). Other E2 basic patch mutants associated with vascular persistence in our experimental viremia model likewise displayed enhanced dissemination following s.c. inoculation (Fig. 4).

Following an s.c. inoculation, viruses and other inoculated material can be transported via the draining lymphatics to the draining lymph node and eventually converge with the circulating blood (39–41). It is generally assumed that enhanced GAG-binding virions, such as WT EEEV, are more easily trapped in the tissue through interactions with GAGs and therefore are expected to disperse poorly compared to variants with lesser affinity for GAGs (5). Indeed, a previous report by Gardner et al. found the EEEV E2 glycoprotein K71/74/77A mutation was associated with enhanced viral signal in the draining lymph nodes at 8 hpi compared to reporter replicons encapsulated with WT EEEV structural proteins (2). Thus, we initially hypothesized that the differences in WT versus K71/74/77A SINV-EEEV dissemination could be driven by the slower initial dispersal of the WT SINV-EEEV from the s.c. inoculation site due to its enhanced GAG-binding properties. However, we found no difference in the initial dispersal of WT and K71/74/77A virions from the s.c. inoculation site to the draining lymph node at 1 hpi (Fig. 5). Instead, our data suggest SINV-EEEV virion-GAG interactions only limit the initial inoculum dispersal following drainage into the circulating blood, whereupon WT virus is depleted from the bloodstream by blood-filtering phagocytes (Fig. 5D). This finding may be due to the sensitivity of our experimental design, which permits early virus detection independent of active viral replication (detection of viral RNA at 1 hpi versus virally expressed luciferase activity at 8 hpi). Our data suggest *in vivo* putative interactions between SINV-EEEV virions and GAGs occur within tissue-specific contexts to affect virion dispersal. This phenotype may be driven by the heterogeneity of GAG abundance and structural features (e.g., degree of sulfation and polymer length, which determine protein-binding [42–44]) across different tissues within individual vertebrate species (45–48).

A point of consideration regarding the EEEV E2 glycoprotein’s basic patches is the potential dual roles of these residues in mediating proteinaceous receptor binding as well as GAG binding. It has recently been shown that the K71/74/77A and K156/R157A mutations severely impair EEEV infection of *Aedes albopictus* mosquitoes (a bridge vector species of EEEV), implicating strong selection from the invertebrate host may maintain these GAG-interaction sites (29). Meanwhile, VLDLR, ApoER2, and LDLR have been identified as cellular receptors for EEEV infection of vertebrate cells (26–28, 49, 50), and several of the basic E2 residues investigated herein are known to mediate interactions with VLDLR (residues K10, K56, K156, R157, K231, and K232) (26–28) and ApoER2 (K156 and R157) (28). It is also possible that EEEV-GAG interactions may directly or indirectly enhance receptor-mediated infection, as a recent preprint shows the K156/R157A and K71/74/77A mutations are associated with decreased infection of mammalian cells over-expressing known EEEV receptors *in vitro* (29). Yet, despite decreased infection seen *in vitro*, these mutant viruses remain capable of productive infection *in vivo* (Fig. 4 and 5) (29), suggesting there may be compensation by other, unknown receptors. Whether the basic patch mutants’ vascular persistence (Fig. 3D) and dissemination (Fig. 4 and 5) phenotypes described herein are driven by decreased affinity for GAGs, diminished binding to proteinaceous receptors, or a synergy between the two is a topic for future investigations.

Another topic for future investigation is whether the location or size of the EEEV E2 basic patches is critical to enhance GAG-binding affinity. Notably, the R84/119 HS-binding site originally identified by cryo-EM (25) is located within the center of the trimeric spike protein (Fig. 1A). We hypothesize that steric hindrance may limit GAG accessibility to this site, and this could account for the intermediate vascular clearance phenotype we observed *in vivo* (Fig. 1C). It is also interesting to note that, like the EEEV R84/119 HS-binding site (Fig. 1A), the enhanced GAG-binding mutations of VEEV TC-83 and CHIKV 181/25, E2-T120R (8, 51), and E2-G82R (15, 52), respectively, are also localized within basic patches in the center of the alphavirus spike. Moreover, examination of the published structures of other alphaviruses (53–59) also predicts basic patches on the surface of their E2 glycoproteins (Fig. S4). While the locations and intensities of these basic patches differ from EEEV, it is interesting to consider whether they may share some of the same functionality in mediating GAG interactions.

In conclusion, this study highlights the strength of the experimental viremia model as a reductionist approach to elucidate virus-host interactions pertinent to viral dissemination. Using this approach, we identified and subsequently corroborated roles for blood-filtering phagocytes and viral E2 glycoprotein basic patches in modulating SINV-EEEV dissemination in an infection model. Furthermore, we found that the E2 glycoprotein also determines initial virion dispersal in an unexpected manner. Our previous work suggests WT SINV-EEEV, but not the K71/74/77A mutant, interacts with host GAGs and blood-filtering phagocytes to promote viral vascular clearance (10). Here we observed no difference in the initial dispersal of WT and K71/74/77A virions from a subcutaneous inoculation site to the draining lymph node. However, upon drainage into the blood circulation, WT but not decreased GAG-binding mutant virions are selectively depleted by blood-filtering phagocytes (Fig. 5). These observations highlight intriguing questions to be answered regarding the role of virion-GAG interactions *in vivo*, particularly the effect of tissue-specific GAGs on viral infection and spread.

## MATERIALS AND METHODS

### Cells and viruses

Sindbis-EEEV (EEEV strain FL93-939) chimeric virus and mutants thereof were generated from cDNA infectious clones. Linearized cDNA infectious clones were used to generate viral RNA using SP6-driven *in vitro* transcription (mMessage mMachine) that was then electroporated into BHK-21 cells (ATCC CCL10). BHK-21 cells were cultured at 37°C in α-Minimum Essential Medium (Gibco) supplemented with 10% fetal bovine serum (FBS), 10% tryptose phosphate broth, and penicillin–streptomycin. Cell culture supernatant was collected at 24–30 hours post-electroporation, clarified, and stored as virus aliquots at −80°C. Plaque assay on BHK-21 cells was used to determine infectious virus titers (60); viral particles were quantified by reverse transcription quantitative PCR (RT-qPCR) of RNA extracted from the virus stocks (10).

The cDNA clones of the WT and E2 glycoprotein mutants K71/74/77A and K156/R157A were a kind gift from William B. Klimstra (University of Pittsburgh). Additional E2 basic patch mutants were generated by site-directed mutagenesis of the WT Sindbis-EEEV infectious clone using the following primers: K71A (5′-CCTACATGAGTTTCATGAACGGCGCAACGCAGAAATCAATAAAGATCG-3′ and 5′-CGATCTTTATTGATTTCTGCGTTGCGCCGTTCATGAAACTCATGTAGG-3′), K74A (5′-TTTCATGAACGGCAAAACGCAGGCATCAATAAAGATCGACAACCTG-3′ and 5′-CAGGTTGTCGATCTTTATTGATGCCTGCGTTTTGCCGTTCATGAAA-3′), K77A (5′-GAACGGCAAAACGCAGAAATCAATAGCTATCGACAACCTGC-3′ and 5′-GCAGGTTGTCGATAGCTATTGATTTCTGCGTTTTGCCGTTC-3′), K71R (5′-GATCTTTATTGATTTCTGCGTTCTGCCGTTCATGAAACTCATGTA-3′ and 5′-TACATGAGTTTCATGAACGGCAGAACGCAGAAATCAATAAAGATC-3′), K74R (5′-GGTTGTCGATCTTTATTGATCTCTGCGTTTTGCCGTTCATG-3′ and 5′-CATGAACGGCAAAACGCAGAGATCAATAAAGATCGACAACC-3′), K77R (5′-GAACGGCAAAACGCAGAAATCAATAAGGATCGACAACCTGC-3′ and 5′-GCAGGTTGTCGATCCTTATTGATTTCTGCGTTTTGCCGTTC-3′), K56A (5′-ATCAGCTATGTTCGGTCTGGCGACGGATGGAGTCGATTTG-3′ and 5′-CAAATCGACTCCATCCGTCGCCAGACCGAACATAGCTGAT-3′), K10A (5′-CGGGCGTGCCAACGCATACTGGGTGAAATGAGTGTCCA-3′ and 5′-TGGACACTCATTTCACCCAGTATGCGTTGGCACGCCCG-3′), R13A (5′-TCAGCAATATACGGGGCTGCCAACTTATACTGGGTGAAATG-3′ and 5′-CATTTCACCCAGTATAAGTTGGCAGCCCCGTATATTGCTGA-3′), K231A (5′-CAGAGTTGTACACCCATTTTGCGTTGTCAATCAGGTAAGCCC-3′ and 5′-GGGCTTACCTGATTGACAACGCAAAATGGGTGTACAACTCTG-3′), K232 (5′-GGGCTTACCTGATTGACAACGCAAAATGGGTGTACAACTCTG-3′ and 5′-GGGCTTACCTGATTGACAACGCAAAATGGGTGTACAACTCTG-3′), R152A (5′-TGCACGCTTGTGAGTGTAAGCGTTACACGGTAATTCAACT-3′ and 5′-AGTTGAATTACCGTGTAACGCTTACACTCACAAGCGTGCA-3′), and R84/119A (set 1: 5′-CAAGGGGCTGAGGTGGCCACATGCAGGTTGTC-3′ and 5′-GACAACCTGCATGTGGCCACCTCAGCCCCTTG-3′; set 2: 5′-CTGTGCACGTATGGGCGTTAGGCCCGTCGT-3′ and 5′-ACGACGGGCCTAACGCCCATACGTGCACAG-3′).

### Animal experiments and virus burden quantification

Four-week-old WT C57BL/6J mice were ordered from The Jackson Laboratory (strain #000664), and LTα-KO mice (breeding pair originally purchased from The Jackson Laboratory, strain #002258) were bred in-house at the University of Colorado Anschutz Medical Campus. All WT mice were age-matched males; LTα-KO mice of both sexes were used; and mice were randomly assigned to experimental groups; prior studies did not identify sex-based differences. All animal experiments were performed in ABSL2 conditions except when CHIKV (ABSL3) was used as an internal control. For phagocyte depletion, CLLs (ClodronateLiposomes.org) were administered i.v. at 10 µL/g on −1 or −2 dpi (20). Phosphate-buffered saline (PBS)-loaded liposomes (ClodronateLiposomes.org) were likewise administered as controls. For virus inoculations, isoflurane vapors were used as anesthesia, and mice were inoculated i.v. with 100 µL of 10^8^ viral genomes diluted in PBS/1% FBS for viral vascular clearance experiments or inoculated s.c. with 10–20 µL of 5 × 10^4^ PFU or 10^7^ viral genomes diluted in PBS/1% FBS for viral dissemination experiments. At indicated timepoints, mice were humanely euthanized and samples were collected.

For serum samples, 20 µL of serum was placed into TRIzol; for foot, brain, and lymph node samples, tissues were placed directly into TRIzol and homogenized using an MP FastPrep 24 (MP Biomedicals). RNA was then extracted from samples using the PureLink RNA Mini Kit (Life Technologies). Virus in samples was quantified by RT-qPCR using SINVnsp2 forward primer (5′-ACGCCGACCGCAACAGTGAG-3′), SINVnsp2 reverse primer (5′-TCTCGCTGCGGACACCC TGA-3′), and SINVnsp2 internal TaqMan probe (5′-ACGTAG TCACCGCTCTTGCCA-3′). As described previously, a standard curve was generated using *in vitro* synthesized RNA; this was used to determine the absolute quantity of viral RNA in experimental samples (60).

### PyMOL modeling

3D renderings of EEEV E2-E1 heterodimer and spike were constructed in PyMOL (version 3.0.3; Schrödinger, LLC) using published structural data, PDB structure 6mx4 (31). Mutagenesis and electrostatic potentials were predicted and mapped using the protein mutagenesis wizard and APBS Electrostatics PyMOL plugin (61), respectively.

## Data Availability

All data presented in this study are available in the Mendeley Data repository: Morrison, Tem (2025), “Basic patches on the E2 glycoprotein of eastern equine encephalitis virus influence viral vascular clearance and dissemination,” Mendeley Data, V1, doi: https://doi.org/10.17632/823tckxwtm.1.

## SUPPLEMENTAL MATERIAL

Supplemental material jvi.00602-25-s0001.pdf Figures S1 to S4.

ASM does not own the copyrights to Supplemental Material that may be linked to, or accessed through, an article. The authors have granted ASM a non-exclusive, world-wide license to publish the Supplemental Material files. Please contact the corresponding author directly for reuse.
