# Supplementary material for: Basic patches on the E2 glycoprotein of eastern equine encephalitis virus influence viral vascular clearance and dissemination in mice
Source: J Virol. 2025 May 19;99(6):e00602-25. doi: 10.1128/jvi.00602-25 (PMC12172491; doi:10.1128/jvi.00602-25)
Supplement: Supplemental material — Figures S1 to S4. [file jvi.00602-25-s0001.pdf]

Supplemental Figures

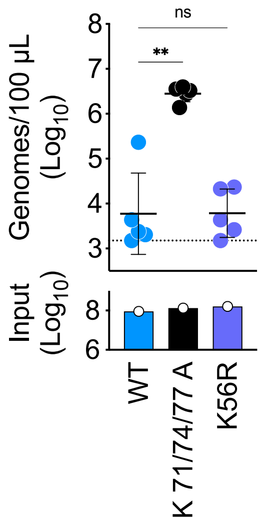

Supplemental Figure 1. Introduction of a positively charged amino acid at position 56 of the SINV-EEEV E2 glycoprotein restores SINV-EEEV vascular clearance. Vascular clearance of SINV-EEEV particles from murine circulation at 3 hpi. WT C57BL/6 mice were inoculated i.v. with 10^8 genomes (input); blood was collected at 3 hpi and viral genomes in the serum quantified. Data is from 1 experiment, 5 mice per virus, and graphed to display mean and standard deviation for each group. Statistics determined by Kruskal-Wallis test followed by Dunn's multiple comparisons test; **, $P<0.01$; ns, not significant.

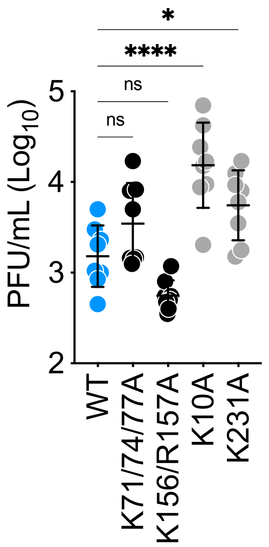

Supplemental Figure 2. Enhanced infectious virus detected in serum of mice s.c. inoculated with SINV-EEEV E2 glycoprotein mutants.

WT C57BL/6 mice were inoculated with 5 x 10^4 PFU of SINV-EEEV in a 20 µL volume by s.c. inoculation into the left rear footpad. Serum was collected at 1 dpi, and infectious virus was quantified by plaque assay on BHK-21 cells. Data is from 2 independent experiments, 4 mice per virus in each experiment, and graphed to display mean and standard deviation for each group. Statistics determined by one-way ANOVA followed by Dunnett's multiple comparisons test; ****, P<0.0001; *, P<0.05; ns, not significant.

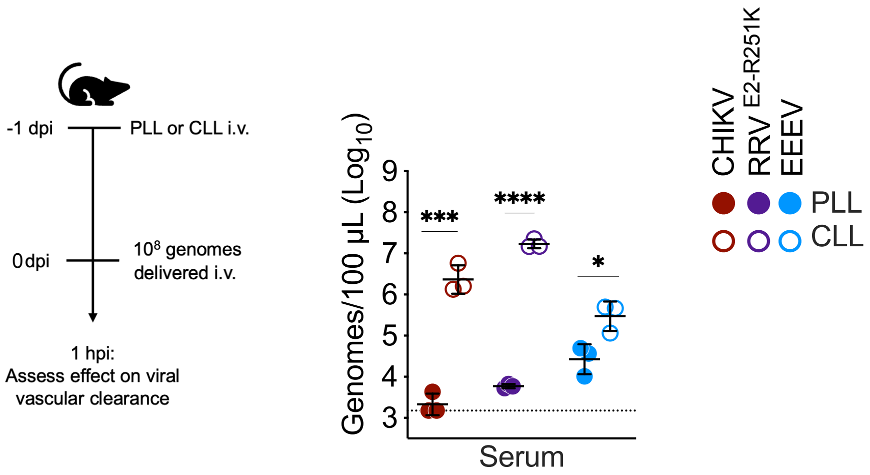

Supplemental Figure 3. Viral vascular clearance by blood-filtering phagocytes is disrupted at 1 day post-CLL treatment.

At -1 dpi, WT mice were treated with CLL or PLL (control) to deplete blood-filtering phagocytes then inoculated i.v. with 10^8 CHIKV, $RRV^{E2\text{-}R251K}$, or SINV-EEEV viral particles (genomes) in a 100 µL volume (*left*). Viral vascular clearance was assessed at 1 hpi (*right*). Data is from 1 experiment, 3 mice per group, and graphed to display each group's mean and standard deviation. Statistics determined by unpaired student's t-test; ****, $P<0.0001$; ***, $P<0.001$; *, $P<0.05$.

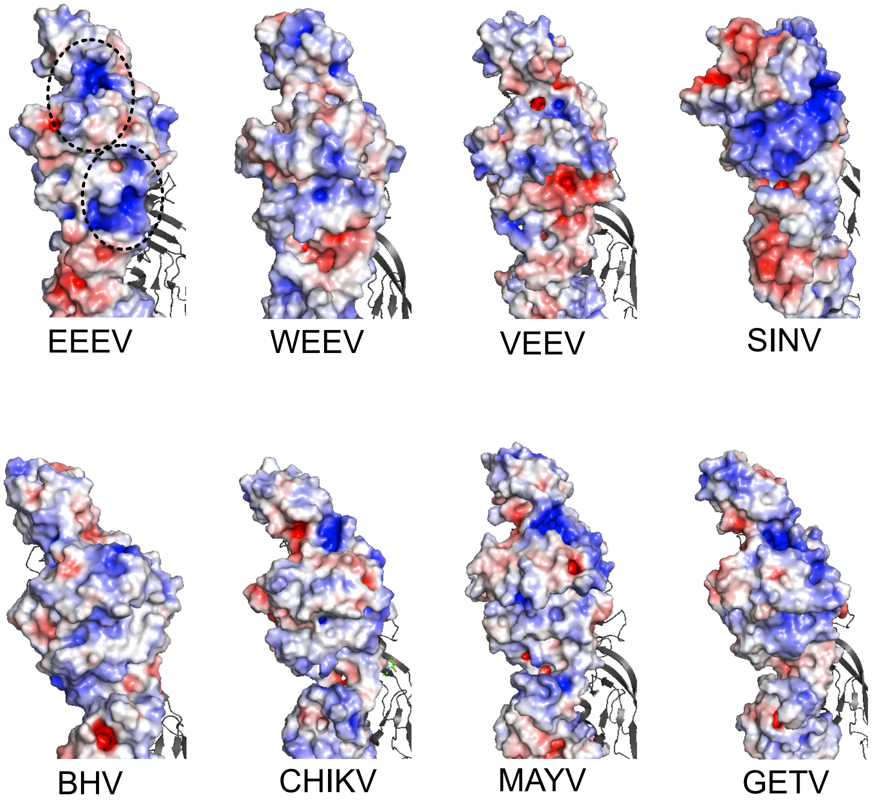

Supplemental Figure 4. Comparison of the electrostatic potentials on the surface of various alphavirus E2 glycoproteins.

Electrostatic potentials on various alphavirus E2 glycoproteins were predicted based on published structures of EEEV (PDB structure 6mx4 (31)), western equine encephalitis virus (WEEV; PDB structure 8daq (53)), Venezuelan equine encephalitis virus (VEEV; PDB structure 7n1i (54)), Sindbis virus (SINV; PDB structure 6imm (55)), Barmah Forest virus (PDB structure 2yew (56)), chikungunya virus (CHIKV; PDB structure 6nk5 (57)), Mayaro virus (MAYV; PDB structure 7ko8 (58)), and Getah virus (GETV; PDB structure 7fd2 (59)). For EEEV, the upper and lower basic patches are indicated in the dashed circles.
